# An intestinal *Candida albicans* model for monomicrobial and polymicrobial biofilms and effects of hydrolases and the Bgl2 ligand

**DOI:** 10.14202/vetworld.2022.1134-1140

**Published:** 2022-04-29

**Authors:** Masfufatun Masfufatun, Rini Purbowati, Nira A. Arum, Mey S. Yasinta, Sri Sumarsih, Afaf Baktir

**Affiliations:** 1 Department of Biochemistry, Faculty of Medicine, University of Wijaya Kusuma Surabaya, Surabaya, Indonesia; 2 Department of Biomedicine and Biomolecular, Faculty of Medicine, University of Wijaya Kusuma Surabaya, Surabaya, Indonesia; 3 Department of Chemistry, Faculty of Science and Technology, Airlangga University, Surabaya, Indonesia; 4 Department of Chemistry, Faculty of Science and Technology, Airlangga University, Surabaya, Indonesia

**Keywords:** *Achatina fulica* hydrolases, Bgl2 ligand, *Candida albicans*, *Escherichia coli*, intestinal polymicrobial biofilm

## Abstract

**Background and Aim:**

*Candida albicans* is the most prevalent human fungal pathogen. In biofilms, *C. albicans* becomes more resistant to antifungal agents because of the production of an extracellular matrix (ECM) that protects the yeast cells. This study aimed to determine the effects of hydrolase enzymes and the Bgl2 ligand on monomicrobial and polymicrobial biofilms.

**Materials and Methods:**

Biofilm induction in rats was carried out using streptomycin (25 mg/kg) and gentamicin (7.5 mg/kg) administered orally once per day for 5 days. Rats were injected subcutaneously with cortisone acetate (225 mg/kg) as an immunosuppressant on day 5. In addition, rats were orally administered *C. albicans* for the single microbial model and a combination of *C. albicans* with *Escherichia coli* for the polymicrobial model. Following the biofilm production, the groups were treated with glucosamine (8.57 mg/kg body weight) and *Achatina fulica* hydrolases (1.5 mL) orally for 2 weeks. The reduction of the biofilm was measured using confocal laser scanning microscopy (CLSM). Data were analyzed using a t-test, with a significance value of 95%.

**Results:**

CLSM images revealed a strong association between *C. albicans* and *E. coli* in the polymicrobial biofilm. On the contrary, the combination treatment using glucosamine and *A. fulica* hydrolases reduced the ECM of the single microbial biofilm (53.58%). However, treatment effectiveness against the matrix (19.17%) was reduced in the polymicrobial model.

**Conclusion:**

There is a strong association between *C. albicans* and *E. coli* in the formation of polymicrobial biofilms. The combination of glucosamine and the *A. fulica* enzyme can reduce the single microbial biofilm ECM; however, it is ineffective in the polymicrobial model.

## Introduction

The human body is a host for one billion microorganisms. *Candida albicans* is usually found in the normal human micro-ecosystem. *Candida* inhabits several body parts of humans, such as the oral cavity, gastrointestinal tract, skin, and vagina [1,2]. The virulence of *C. albicans* is related most commonly to the formation of biofilms. A biofilm is a structured microbial community covered in an extracellular matrix (ECM) and attached to a surface. The biofilm shows phenotypic characteristics different from planktonic or motile cells [3]. In the biofilm form, there is significantly greater resistance to antimicrobial agents [4-8]. The ECM is secreted by the sessile communities and protects them. It consists mainly of polysaccharides, such as glucans, mannan, and chitin, and it acts as a barrier and hampers the ability of antifungal molecules to reach the cells. However, it is not the only cause of biofilm resistance [5,9,10].

As the presence of the ECM can inhibit antifungal action, it is essential to develop research to find herbal remedies that can destroy the matrix material. Enzymatic treatment may be a good strategy to improve antifungal performance. In our previous work, we successfully inhibited biofilm formation using the Bgl2p ligand, glucosamine, as an inhibitory molecule. Bgl2p is a glucosyltransferase encoded by the Bgl2 gene. This glucosyltransferase is responsible for forming covalent bonds between 1,3-glucan and other cell wall components and ECM components [11]. In this work, we combined the action of the glucosamine ligand and the hydrolase mixture produced by *Achatina fulica* to support antimicrobial performance for hydrolysis of the ECM in the monomicrobial and polymicrobial models of *C. albicans*.

Therefore, the purposes of this study were (1) to prove the ability of *C. albicans* to form a mixed or polymicrobial intestinal biofilm with *Escherichia coli*; (2) to evaluate the interactions between *C. albicans* and *E. coli* in biofilms from rat models; and (3) to investigate the performance of glucosamine (as the Bgl2 ligand) and the combination of *A. fulica* hydrolases against single and polymicrobial biofilms.

## Materials and Methods

### Ethical approval

All animal experiments were carried out in Laboratory Animals, Faculty of Veterinary, Universitas Airlangga, Surabaya, Indonesia. Before the animal experiments were conducted, the ethical clearance (Reg. 271-KE) was obtained from the Animal Care and Use Committee, Faculty of Veterinary Medicine Universitas Airlangga.

### Study period and location

This study was conducted from March to November 2021. The experiment using animals was managed in Animal Laboratory, Faculty of Veterinary Medicine, Universitas Airlangga. Meanwhile, the observation of confocal laser scanning microscope (CLSM) was carried out in Central Laboratory of Biological Sciences, Universitas Brawijaya.

### Microbial strains, media, and growth conditions

The strains used in this study were *C. albicans*-ATCC 10231 and *E. coli-*ATCC 25992 purchased from the Balai Besar Laboratorium Kesehatan. Overnight *C. albicans* cultures were grown in yeast extract–peptone–dextrose (YPD) medium (1% yeast extract [BD Biosciences, USA], 2% peptone [Oxoid Ltd, UK], and 2% dextrose [Conda Pronadisa, Spain]) at 30°C. Overnight *E. coli* cultures were grown in Luria–Bertani (LB) medium (1% tryptone [Himedia, India], 0.5% yeast extract, and 1% NaCl [Merck, Germany]) at 37°C.

### Study design

The animals used for the *in vivo* experiments were male Wistar rats weighing about 200 g. All animals were acclimatized for 1 week and given a standard *ad libitum* diet. The rats were divided into five treatment groups, each consisting of five individuals: Group 1, negative control (without biofilm induction); Group 2, monomicrobial biofilm control; Group 3, biofilm treatment with enzyme and antifungal for monomicrobial film; Group 4, polymicrobial biofilm control; and Group 5, biofilm treatment with enzyme and antifungal for polymicrobial film.

### *In vivo* monomicrobial induction of *C. albicans* biofilm

Biofilm induction in rat models was carried out according to Masfufatun *et al*. [12] methods, with modifications. The *C. albicans* stock culture was grown on Sabouraud dextrose agar (SDA; 1% peptone, 4% dextrose, and 1.5% agar) at room temperature (25°C). A single colony was inoculated in YPD broth, with shaking at 120 rpm (37°C) overnight. The inoculum was then orally administered to rats in the biofilm groups. During the biofilm induction periods, rats were given aminimum portion of food with 2.5 mL of spider medium (1% nutrient broth, 1% D-mannitol [Himedia], and 0.2% monopotassium phosphate [Merck]) twice per day to induce biofilm formation further. Rat models from Groups 2 and 4 were sacrificed to observe the biofilm of *C. albicans* macroscopically on the mucous membrane of the cecum after biofilm induction, 35 days after the inoculation of C. *albicans*.

### *In vivo* polymicrobial induction of *C. albicans–E. coli* biofilms

The induction of the polymicrobial model was carried out using methods similar to those for the *in vivo* monomicrobial model, with the addition of orally administered *E. coli* inoculum in LB medium along with *C. albicans*.

### Enzymatic treatments with Bgl2 ligand and antimicrobial combination

After the biofilm induction phase, Groups 3 and 5 received the combination of the enzymatic Bgl2p ligand and antimicrobial treatment for 1 week. The combination of treatment methods is described below. The *A. fulica* enzyme mixture was harvested according to Baktir *et al*. [13]. The harvested enzyme mixture (1.5 mL) and glucosamine (8.57 mg/kg body weight [BW] and fluconazole, and 6 mg/kg BW; with the addition of tetracycline for the polymicrobial model) were orally administered to the model rats of Groups 3 and 5. The rats were then sacrificed after the treatment period to macroscopically observe the *C. albicans* biofilm on the mucous membrane of the cecum after biofilm induction.

### Macroscopic identification of biofilm

The intestinal model biofilm was identified by macroscopic observations of the mucous membrane of the cecum after dissecting and photographing it with a camera (Canon EOS m3).

### CLSM

The sample preparation was prepared using the following protocol. Clean and sliced cecum samples were fixed in 10% formalin buffer, submerged in paraffin, and cut into sections of 5-μm thickness. The cecum tissues were deparaffinized with xylol (2 times) for 10 min each, hydrated with ethanol (absolute ethanol, 90% and 70%, consecutively), washed with phosphate-buffered saline (PBS) for 5 min, blocked with 2% bovine serum albumin (BSA) in PBS at 25°C for 1 h, and rewashed with PBS for 8 min. A 100-μg/mL aliquot of Concanavalin A (con A; bioWORLD, USA) was dropped onto the sample, which was then incubated at 25°C for 1 h. Finally, the samples were rewashed with PBS, reblocked with 2% BSA in PBS, and observed under a confocal microscope (Olympus FV1000).

### Statistical analysis

All data were subjected to an independent t-test to assess significant differences between two groups. Data normality was calculated using the Shapiro–Wilk test (for samples <50). Statistical analyses were performed using Statistical Package for the Social Sciences 16.0 software (IBM Corp., NY, USA). A value of p<0.05 was considered to indicate a significant difference between the control and treatment groups. All values are expressed as the mean±standard deviation.

## Results

### Macroscopic and microscopic observations of monomicrobial and polymicrobial *C. albicans* biofilms in an *in vivo* rat model

**Figure-1:**
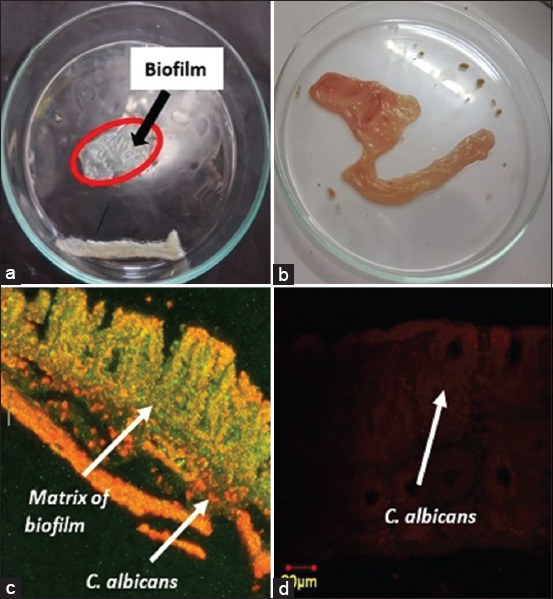
Macroscopic and microscopic image of monomicrobial *Candida* *albicans* biofilm in ceccum mucous membrane. Macroscopic: (a) Biofilm of the Group 2 rat model and a (b) Normal rat model (Group 1) (b). Biofilm shown in the red circle, appeared as white lesions. Microscopic: (c) Biofilm of the Group 2 rat model and (d) Normal rat model (Group 1). The green colored image displayed a matrix, colored using Concavaline A. The red colored image displayed *C. albicans* cells, colored using polyclonal-antibody anti-*Candida* conjugated to Tetramethyl Rhodamine Isothiocyanate. Yellow-orange colored displayed the superimposed image of *C. albicans* and matrix confocal image.

The *in vivo* monomicrobial *C. albicans* biofilms in the rats of Group 2 appeared as white lesions in the mucous membrane of the cecum under macroscopic evaluation Figure-1a. The normal rat model (Group 1) showed a normal mucous membrane of the cecum without white lesions Figure-1b. Microscopic images of *C. albicans* and the matrix of the monomicrobial biofilm model of the biofilm control group (Group 1) (c) and control group (Group 2) (d) are shown. The green and red intensities in the biofilm group (Figure-1c) were stronger than those in the control group (Figure-1d). Green fluorescence indicates the presence of *C. albicans* biofilm matrix, and red luminescence indicates the presence of *C. albicans* cells. Yellow fluorescence indicates the presence of *C. albicans* cells in the biofilm covered with the resulting ECM.

**Figure-2:**
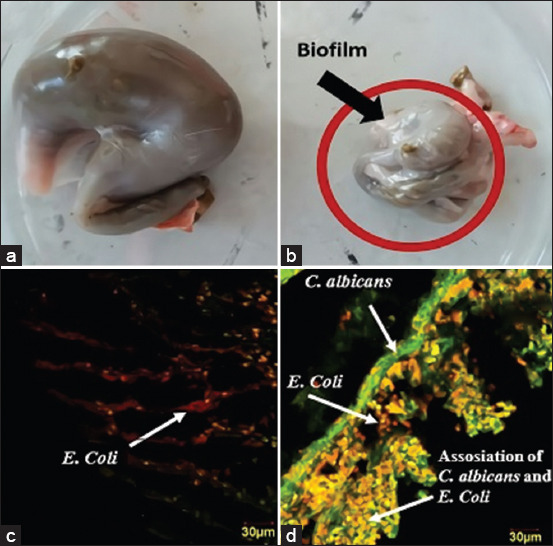
Macroscopic and microscopic image of polymicrobial *Candida albicans* biofilm in cecum mucous membrane. Macroscopic: (a) Normal rat model (Group 1) and (b) Polymicrobial biofilm of the Group 4 rat model. Biofilm shown in the red circle, appeared as white lesions. Microscopic: (c) Normal rat model (Group 1) and (d) polymicrobial biofilm of the Group 4 rat model. The green colored image displayed *C. albicans* cells, colored using polyclonal-antibody anti-*Candida*-fluorescein isothiocyanate. The red colored image displayed *Escherichia coli* cells, colored using SYTO-59. Yellow-orange colored displayed the superimposed image of *C*. *albicans* and *E. coli* confocal image.

The *in vivo* polymicrobial biofilm model of Group 4 appeared as white lesions located in the mucous membrane of the cecum, as presented in Figure-2b. The normal rat model showed a normal outer mucous membrane of the cecum without white lesions, as presented in Figure-2a. There was a considerable difference in the cecum size between the normal group and the Group 4 models. Yellow fluorescence in the biofilm group (Figure-2d) is the result of overlapping red (*E. coli*) and green (*C. albicans*) images, showing that *C. albicans* cells interacted with bacterial cells to form polymicrobial biofilms. In the control group (Figure-2c), a portion was red with moderate intensity, proving that there were still many bacteria in the digestive tract.

### CLSM analysis of monomicrobial and polymicrobial *C. albicans* biofilms in an *in vivo* rat model before and after treatment

**Figure-3:**
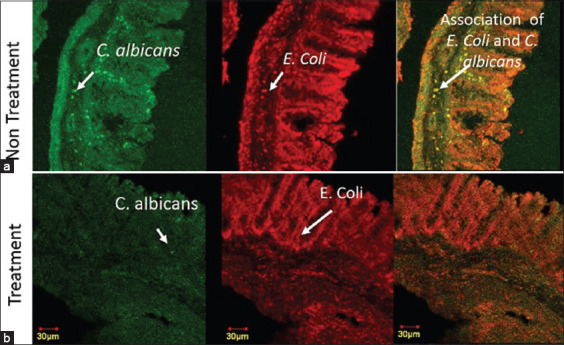
Confocal laser scanning microscopy image of *Candida albicans* and *Escherichia* *coli* cells. Confocal image of *C. albicans*-*E. coli* polymicrobial biofilm model. (a) Non-treatment polymicrobial biofilm group. (b) Treatment polymicrobial biofilm group. The green colored image displayed *C*. *albicans* cells, colored using polyclonal-antibody anti-Candida-fluorescein isothiocyanate. The red colored image displayed *E. coli* cells, colored using SYTO-59. Yellow-orange colored displayed the superimposed image of *C. albicans* and *E. coli* confocal image.

**Figure-4:**
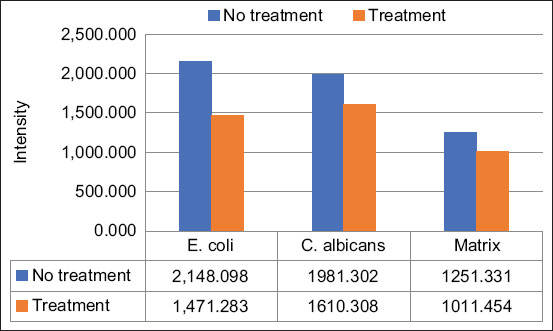
Quantitative data from confocal laser scanning microscopy assessment in polymicrobial biofilm model. The mean intensities of *Escherichia coli*, *Candida albicans*, and matrix were shown in the data below the chart. All of the *E. coli*, *C. albicans*, and matrix consecutively shown the decreased intensities in the treatment group by 31.51%, 39.03%, and 19.17%. **indicates the significant differences (p<0.05)

Confocal imaging was used to assess the association between *C. albicans* and *E. coli* from the same polymicrobial model sample with different fluorescences. The confocal images of *C. albicans* and *E. coli* showed an association between these two species, as *C. albicans* mainly appeared in the same location as *E. coli*, as seen from the yellow–orange superimposed image (Figure-3a). The treatment resulted in a decrease in fluorescence intensity, as evidenced by the dimmer appearance in the confocal image of the treatment group or Group 2 (Figure-3b) and the quantitative data of the mean intensity that showed a decrease in the treatment group (Figure-4). The decreased intensity was proportional to the decreased number of cells, as the fluorescence appeared due to the specific binding of the dye with parts of the cells.

**Figure-5:**
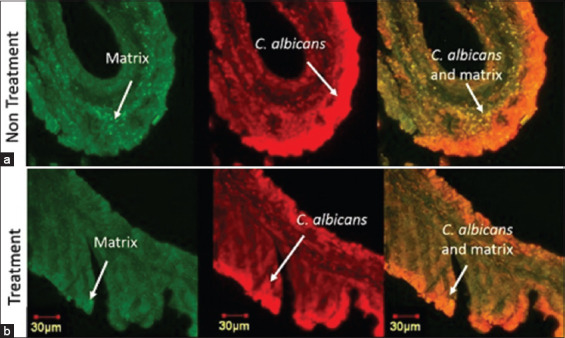
Confocal laser scanning microscopy image of *Candida* *albicans* cells and matrix of the polymicrobial biofilm model. (a) Non-treatment polymicrobial biofilm group. (b) Treatment polymicrobial biofilm group. The green colored image displayed matrix, colored using Concanavalin A. The red colored image displayed *C. albicans* cells, colored using polyclonal-antibody anti-*Candida* conjugated to TRITC. Yellow-orange colored displayed the superimposed image of *C. albicans* and matrix confocal image.

The effect of *C. albicans* cells and matrix treatment in the polymicrobial model was also observed with confocal imaging. The treatment decreased *C. albicans* cells and matrix, demonstrated by the lower intensities of the confocal image fluorescence (Figure-5b), than that of the non-treatment group (Figure-5a). The decrease in intensity was proportional to the decrease in the ECM (Figure-4).

**Figure-6:**
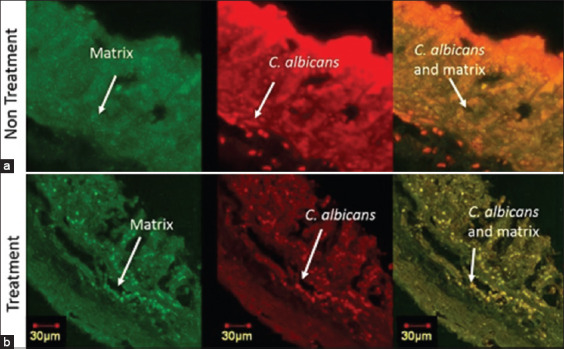
Confocal laser scanning microscopy image of *Candida albicans* cells and matrix of the monomicrobial biofilm model. (a) Non-treatment monomicrobial biofilm group. (b) Treatment monomicrobial biofilm group. The green colored image displayed matrix, colored using Concanavalin A. The red colored image displayed *C. albicans* cells, colored using polyclonal-antibody anti-*Candida* conjugated to TRITC. Yellow-orange colored displayed the superimposed image of *C. albicans* and matrix confocal image.

**Figure-7:**
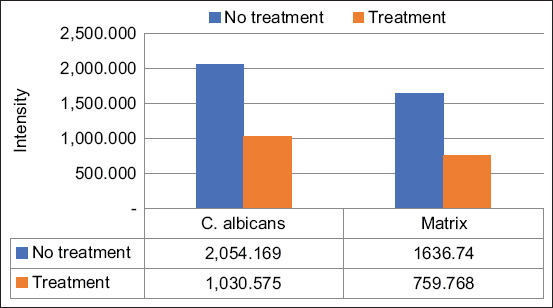
Quantitative data from confocal laser scanning microscopy (CLSM) assessment in polymicrobial biofilm model. Quantitative data from CLSM assessment in monomicrobial biofilm model. The mean intensities of *Candida albicans* and matrix were shown in the data below the chart. Both of the *Candida albicans* and matrix consecutively shown the decreased intensities in the treatment group by 49.83% and 53.58%. **Indicates the significant differences (p<0.05).

For the monomicrobial model, the results agreed with the polymicrobial biofilm model. A dimmer confocal image (Figure-6) demonstrated that the *C. albicans* cells and matrix had reduced intensities in the treatment group. Quantitatively, the monomicrobial biofilm had a smaller number of cells and matrix (indicated by the lower intensities) than the polymicrobial biofilm of the treatment group (Figure-7).

## Discussion

The biofilm lifestyle protects cells that reside within the thick layer of the ECM. The biofilm allows cells to develop up to 1000 times more resistance to antimicrobials than planktonic cells, leaving biofilm eradication a challenging task [14,15]. For *C. albicans*, the biofilms grown on surfaces show significantly increased minimum inhibitory concentrations to antifungals, including fluconazole, compared to planktonic cells [15,16].

The biofilm of *C. albicans* consists of blastospore-type cells as the basal layer and a superficial layer of ECM and hyphal-type cells. The ECM comprises carbohydrates (glucose, N-acetylglucosamine, mannose, and rhamnose), proteins, phosphorus, uronic acid, and hexosamine [9,17]. One of the carbohydrate components is β-1,3-glucan, a glucose polymer, thought to be the main ECM component in biofilms. β-1,3-Glucans are synthesized by glucan synthase, a membrane-bound protein, using uridine diphosphate glucose as a substrate and secret the molecules to the ECM. This component in *C. albicans* biofilm cells may contribute to resistance to antifungal drugs. Some studies have reported that β-1,3-glucans can be found in the supernatant around the biofilm and in the matrix. The level of this substance increased to a high concentration during the biofilm formation phase. The other evidence is that enzymatic treatment of β-1,3-glucans using β-1,3-glucanase at high concentrations eliminates biofilms but does not significantly affect planktonic cells. Enzymatic treatment using β-1,3-glucanase at low concentrations poorly disrupts biofilms; however, in combination with fluconazole, it improves the performance of the antifungal [18-20]. A previous study by Nett *et al*. [20] also suggested that β-1,3-glucans bind to fluconazole in biofilms, decreasing the antifungal efficacy to control biofilm-associated cells. Therefore, disrupting the β-1,3-glucan component using β-1,3-glucanase is a good strategy to support antifungal effectiveness.

The roles of several genes in the biofilm formation process have been investigated. The glucan transferases Phr1p and Bgl2p, encoded by *PHR1* and *BGL2*, respectively, and exoglucanase, encoded by *XOG1*, are predicted to exist in the ECM and have the roles of delivering and accumulating β-1,3-glucans in the ECM. Mutant strains lacking these genes show more susceptibility to fluconazole [21]. Therefore, the competitive inhibition of enzymes responsible for biofilm construction, such as Bgl2p, can be an effective way to promote the antimicrobial potential for killing cells directly.

*A. fulica* is a natural source of several hydrolase enzymes. It produces the enzyme mixture through the digestive gland to help digest food. However, several nondigestible materials are digested by the microbial enzymes produced in their gastrointestinal tract. The enzyme mixtures of the digestive tract of this species are carbohydrases, such as mannosidase, glucosidase, chitinase, b-glucanases, proteinase, and lipase [22-25]. The utilization of *A. fulica* might reduce the cost of enzyme production, as this species is fairly abundant, especially in a tropical and humid area like Indonesia. The enzymes from *A. fulica* can successfully lyse the fungal cell wall of *Candida* spp. [26]. *Candida* spp. cell walls have components similar to the ECM, with β-1,3-glucans and b-1,6-glucan being the major carbohydrate components (50%–60%). Therefore, the enzyme mixtures of *A. fulica* have great potential as an affordable and effective antibiofilm treatment, as supported by Nett *et al*. [20], wherein treatment with β-1,3-glucanase was highly effective in eradicating *C. albicans* biofilms.

**Figure-8:**
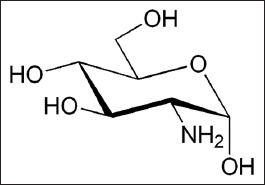
The structure of glucosamine.

Therefore, we hypothesized that the combination of treatment using *A. fulica* mixture hydrolases and the *Bgl2* ligand, the glucose substrate look-alike, glucosamine (Figure-8), could work synergistically to enhance the eradication of the ECM and increase the killing of pathogenic cells that reside within the ECM. The matrix was decreased through hydrolysis of its complex polymer components, mainly β-1,3-glucan, to oligomers or monomers. The ligand, glucosamine, binds to the specific binding site of glucan transferase, replacing its original substrate, glucose, and inhibits β-1,3-glucan delivery and the accumulation of β-1,3-glucan in the matrix, thus further disrupting biofilm formation. However, in this study, we did not compare the performance of this potential antibiofilm as a combination and as separate components. We compared the action of this combined treatment with the results of the *in vivo* application of the monomicrobial and polymicrobial biofilm model.

Biofilms consisting of single microbial species have been extensively studied; however, more recent investigations have found that polymicrobial biofilms are the dominant form in nature. This study investigated the interaction between bacteria and fungi in a polymicrobial biofilm model composed of *E. coli* and *C. albicans*. Both species are predominant pathogens that can grow as biofilms in medical devices [2]. This study demonstrated that ECM disruption using a ligand–enzyme combination treatment in the monomicrobial biofilm model was more effective than the treatment in the polymicrobial biofilm model. The ECM was decreased significantly by 53.58% in the monomicrobial model. However, in the polymicrobial model, the treatment decreased the matrix intensity by 19.17%; this decrease was also significant (Figures-4 and 7). The difference in the ligand–enzyme performance against the matrix material might be because of the strong and synergistic association between *E. coli* and *C. albicans*. A previous study stated that *E. coli* might facilitate the attachment of *C. albicans* to the host surface, resulting in stronger binding between the cells and the host [27,28].

## Conclusion

Treatment targeting matrix hydrolysis and the inhibition of Bgl2 using a combination of antimicrobials successfully reduced the biofilm matrix of *E. coli* and *C. albicans*. In this study, we have found a mixture of glucosamine and Bgl2 enzyme as herbal remedies to destroy the matrix material of *C. albicans* biofilm in the monomicrobial model. However, it showed limitated activity towards polymicrobial biofilm of *C. albicans* and *E. coli*. In the next study we will explore antibiofilm especially for *E.coli*.

## Authors’ Contributions

MM: Conceived and designed the study, performed the experimental work, and reviewed the manuscript. NAA and MSY: Collected microorganism isolates and reviewed the manuscript. AB and RP: Data interpretation, statistical study, and reviewed the manuscript. SS: Conducted the literature review and drafted the manuscript. All authors have read and approved the final manuscript.
